# Supplementary material for: Splice-Junction-Based Mapping of Alternative Isoforms in the Human Proteome
Source: Cell Rep. Author manuscript; Available in PMC 2020 Jan 15. (PMC6961840; doi:10.1016/j.celrep.2019.11.026)
Supplement: 2 [file NIHMS1546469-supplement-2.zip › DF1/PXD010154/Lung/Lung_8_DRAP1_VAVEEGLPGDPVAER.pdf]

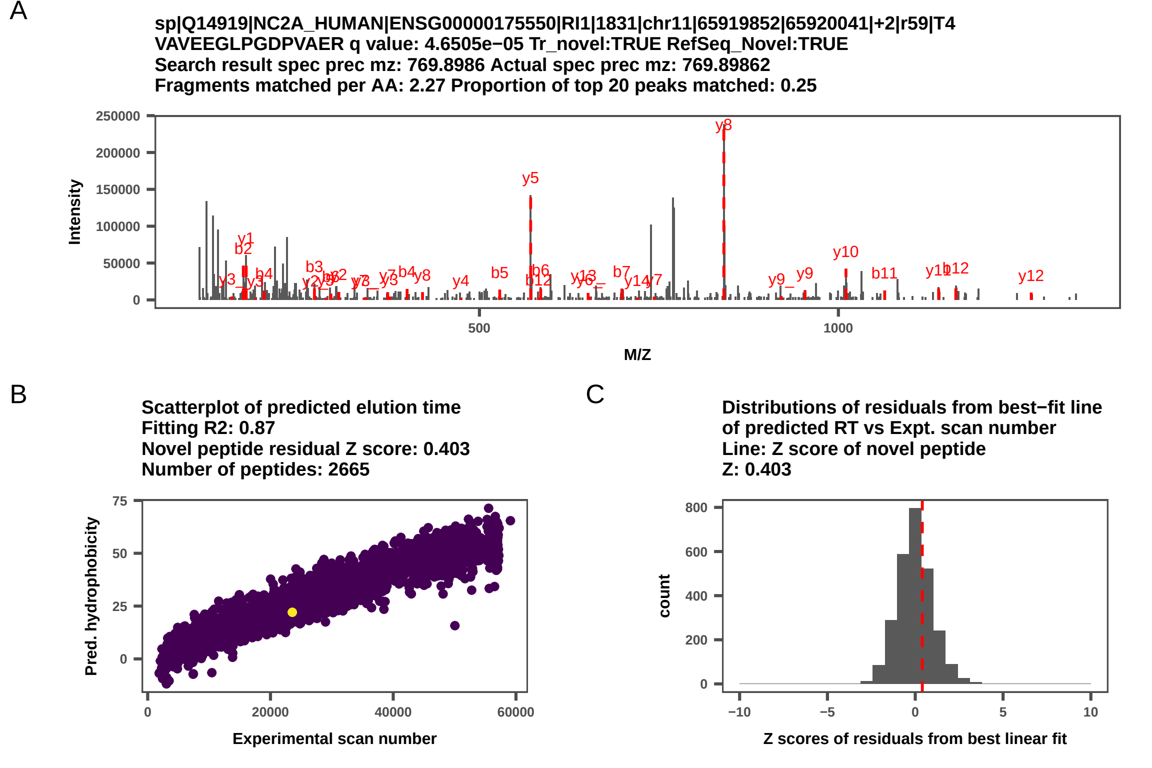

A
sp|Q14919|NC2A_HUMAN|ENSG00000175550|RI1|1831|chr11|65919852|65920041|+2|r59|T4
VAVEEGLPGDPVAER q value: 4.6505e−05 Tr_novel:TRUE RefSeq_Novel:TRUE
Search result spec prec mz: 769.8986 Actual spec prec mz: 769.89862
Fragments matched per AA: 2.27 Proportion of top 20 peaks matched: 0.25
Intensity
250000
200000
150000
100000
50000
0
y8
y5
y1
b2
b3
b4
y4
b5
b6
b12
b7
y9
y10
b11
b12
y12
500
1000
M/Z
B
Scatterplot of predicted elution time
Fitting R2: 0.87
Novel peptide residual Z score: 0.403
Number of peptides: 2665
Pred. hydrophobicity
75
50
25
0
0
20000
40000
60000
Experimental scan number
C
Distributions of residuals from best−fit line
of predicted RT vs Expt. scan number
Line: Z score of novel peptide
Z: 0.403
count
800
600
400
200
0
−10
−5
0
5
10
Z scores of residuals from best linear fit
